# Current HHT genetic overview in Spain and its phenotypic correlation: data from RiHHTa registry

**DOI:** 10.1186/s13023-020-01422-8

**Published:** 2020-06-05

**Authors:** Rosario Sánchez-Martínez, Adriana Iriarte, José María Mora-Luján, José Luis Patier, Daniel López-Wolf, Ana Ojeda, Miguel Angel Torralba, María Coloma Juyol, Ricardo Gil, Sol Añón, Joel Salazar-Mendiguchía, Antoni Riera-Mestre, for the RiHHTa Investigators of the Rare Diseases Working Group from the Spanish Society of Internal Medicine, C. Alonso-Cotoner, S. Añón, M. Beneyto, M. M. Bermejo-Olano, P. Cerdà, F. Cruellas, A. De Los Santos, L. Díez, A. Fernández, J. S. García-Morillo, R. Gil, J. F. Gómez-Cerezo, V. Gómez del Olmo, A. González-García, A. Iriarte, P. Iglesias, M. C. Juyol, N. López-Osle, M. López, D. López-Wolf, J. M. Mora-Luján, M. Moreno, A. Ojeda, J. L. Patier, J. A. Pérez de León, M. L. Perez, A. Riera-Mestre, S. Rivera, S. Rodríguez, R. Sánchez-Martínez, M. A. Torralba, R. Zarrabeitia

**Affiliations:** 1 Internal Medicine Department, Hospital General Universitario de Alicante - ISABIAL, Alicante, Spain; 2 Rare Diseases Working Group, Spanish Society of Internal Medicine, Madrid, Spain; 3 Hereditary Hemorrhagic Telangiectasia Unit, Internal Medicine Department, Hospital Universitari de Bellvitge – IDIBELL, Feixa Llarga s/n. 08907 L’Hospitalet de Llobregat, Barcelona, Spain; 4 Department of Internal Medicine, Systemic and Orphan Diseases Unit, University Hospital Ramón y Cajal, University of Alcalá, IRYCIS, Madrid, Spain; 5 Internal Medicine Department, Hospital Universitario Fundación Alcorcón, Madrid, Spain; 6 Internal Medicine Department, Hospital Insular Universitario de Gran Canaria, Gran Canaria, Spain; 7 Internal Medicine Department, Hospital Clínico Universitario Lozano Blesa, Zaragoza, Spain; 8 Internal Medicine Department, Hospital Universitario Miguel Servet, Zaragoza, Spain; 9 Internal Medicine Department, Hospital La Fe, Valencia, Spain; 10 Internal Medicine Department, Hospital Arnau de Vilanova, Valencia, Spain; 11 Health in Code, A Coruña, Spain; 12 Clinical Genetics Program, Hospital Universitari de Bellvitge – IDIBELL, Barcelona, Spain; 13 Genetics Department, Universitat Autònoma de Barcelona, Barcelona, Spain; 14 Faculty of Medicine and Health Sciences, Universitat de Barcelona, Barcelona, Spain

**Keywords:** Hereditary hemorrhagic telangiectasia, Genetic test, Phenotype, Genotype, Rare diseases

## Abstract

**Background:**

Hereditary hemorrhagic telangiectasia (HHT) is a rare vascular disease with autosomal dominant inheritance. Disease-causing variants in endoglin (*ENG*) and activin A receptor type II-like 1 (*ACVRL1*) genes are detected in more than 90% of cases submitted to molecular diagnosis.

**Methods:**

We used data from the RiHHTa (Computerized Registry of Hereditary Hemorrhagic Telangiectasia) registry to describe genetic variants and to assess their genotype-phenotype correlation among HHT patients in Spain.

**Results:**

By May 2019, 215 patients were included in the RiHHTa registry with a mean age of 52.5 ± 16.5 years and 136 (63.3%) were women. Definitive HHT diagnosis defined by the Curaçao criteria were met by 172 (80%) patients. Among 113 patients with genetic test, 77 (68.1%) showed a genetic variant in *ACVRL1* and 36 (31.8%) in *ENG* gene. The identified genetic variants in *ACVRL1* and *ENG* genes and their clinical significance are provided. *ACVRL1* mutations were more frequently nonsense (50%) while *ENG* mutations were more frequently, frameshift (39.1%). *ENG* patients were significantly younger at diagnosis (36.9 vs 45.7 years) and had pulmonary arteriovenous malformations (AVMs) (71.4% vs 24.4%) and cerebral AVMs (17.6% vs 2%) more often than patients with *ACVRL1* variants. Patients with *ACVRL1* variants had a higher cardiac index (2.62 vs 3.46), higher levels of hepatic functional blood tests, and anemia (28.5% vs 56.7%) more often than *ENG* patients.

**Conclusions:**

*ACVRL1* variants are more frequent than *ENG* in Spain. *ACVRL1* patients developed symptomatic liver disease and anemia more often than *ENG* patients. Compared to *ACVRL1*, those with *ENG* variants are younger at diagnosis and show pulmonary and cerebral AVMs more frequently.

## Introduction

Hereditary hemorrhagic telangiectasia (HHT) or Rendu-Osler-Weber syndrome (ORPHA774; HHT 1: OMIM# 187300; HHT 2: OMIM# 600376) is a rare autosomal-dominant multisystemic vascular disease characterized by telangiectasia and larger vascular malformations (VMs), with a prevalence of 1 in 6000 [1–4]. Although more than 800 pathogenic variants in more than 5 genes have been reported, disease-causing variants in endoglin (*ENG*; chromosome 9q) and activin A receptor type II-like 1 (*ACVRL1*; chromosome 12q) genes are detected in more than 90% of cases submitted to molecular diagnosis and cause HHT1 and HHT2, respectively [5–7]. Mutations in *SMAD4* (encoding the transcription factor Smad4) have been described in less than 2% of the HHT population and cause juvenile polyposis/HHT overlap syndrome [8]. Endoglin (encoded by *ENG*) is an auxiliary co-receptor at the endothelial cell surface that promotes BMP9 signaling through the activin receptor-like kinase 1 (ALK1; encoded by *ACVRL1*). Both proteins contribute to the signaling hub formed by BMP9-Endoglin-ALK1-Smad with a high impact in angiogenesis [9–11].

HHT can be diagnosed either through molecular genetic testing or using the Curaçao clinical criteria (recurrent epistaxis, cutaneous/mucosal telangiectasia, visceral involvement and a first-degree relativewith HHT) [4, 5, 7, 12]. However, since HHT is an age-dependent disease, genetic study is a very useful tool for diagnosis in young patients [4, 5, 7]. Telangiectasia is the characteristic lesion in HHT and shows dilated postcapillary venules directly connected with dilated arterioles losing the capillary bed [7, 13]. Telangiectasia in nasal mucosae causes spontaneous and recurrent epistaxis that are the most common and usually the earliest clinical manifestation of HHT [7, 12, 14]. Although endoglin and ALK1 are components of the same BMP9 receptor complex, they are structurally and functionally different proteins. Thus, pathogenic variants in their genes are related to different clinical phenotypes [5, 9]. Pulmonary arteriovenous malformations (AVMs) and brain VMs are more common in patients with HHT1, while vascular hepatic malformations are more frequent in HHT2 [4, 7, 15].

Of note, there is high inter-familial as well as intra-familial vascular involvement and clinical manifestations variability [7, 16]. Therefore, genetic study is essential for diagnostic confirmation and also for relatives’ genetic screening [5, 7, 17]. This low prevalence and clinical variability makes HHT understanding challenging, especially in uncommon situations [18]. Development of registries that include clinical and genetic data may help to overcome these difficulties, and represent a priority in the management of rare diseases [19, 20]. The use of quality registries reflects patients’ management in standard clinical practice, helps reaching a consensus on diagnostic-therapeutic measures and also generates hypotheses for developing clinical trials [19–21]. The aim of the present study is to describe the genetic variants and their clinical correlation in HHT patients from the RiHHTa (Computerized Registry of Hereditary Hemorrhagic Telangiectasia) registry, comprehending a cohort of individuals followed-up at specialized HHT centers in Spain [14].

## Material and methods

### Study design

RiHHTa is a multicenter, prospective and observational registry, developed within the Rare Diseases Working Group of the Spanish Society of Internal Medicine. RiHHTa registry is currently formed by 42 researchers from 29 Spanish hospitals. RiHHTa has an online design (accessible from https://rihhta.healthincode.com) available in Spanish or English with individual encoded access for each researcher. The rationale and methodology of RiHHTa have been published elsewhere [14]. Patients’ personal data included in the registry are in agree with Spanish personal data law (Ley 15/1999 de Protección de Datos de Carácter Personal). To safeguard the identity of the included patients, the RiHHTa registry generate an encrypted identifier for each patient. All patients (or their relatives) provided written consent for participation in the registry. The design of the RiHHTa registry was approved by the Ethics Committee of the Hospital Universitari de Bellvitge (Barcelona, Spain; ethic approval number PR241/16). The creation of RiHHTa counted on the nonremunerated collaboration of the genetic studies company Health in Code (A Coruña, Spain), which has broad experience in cardiomyopathy registries [22].

The main objective of the present study was to describe the type of genetic variants identified in HHT patients included in RiHHTa registry and to analyse the genotype-phenotype correlation according to these mutations in *ENG* or *ACVRL1* genes.

### Patient and variables

Patients included in the RiHHTa registry with a “definite” clinical diagnosis according to the Curaçao Criteria (≥ 3 criteria) or with a genetic diagnosis were considered for this study [12]. The inclusion period was from June 2016 to June 2019. Demographic data, clinical characteristics and complementary tests were collected according to mutations in *ENG* or *ACVRL1* genes. The severity of nosebleeds was measured according to the epistaxis severity score (ESS). ESS is an on-line tool that estimates the severity of epistaxis according to clinical data occurring during the last 3 months. Epistaxis is considered moderate or severe if ESS results > 4 or > 7 points, respectively [23].

For the screening of pulmonary visceral involvement, a contrast transthoracic echocardiography (TTE) was performed to establish the degree of right-left shunt (R-L) and the need to undergo a thoracic computed tomography (CT) angiography to objectively confirm the presence of pulmonary arteriovenous malformations (AVM) [7, 24]. In addition, an abdominal CT angiography, magnetic resonance imaging (MRI) or doppler ultrasound were performed to study hepatic and/or abdominal VMs. Hepatic involvement was defined according to the three classical patterns of abnormal vascular communications: arteriovenous shunt (from hepatic artery to hepatic vein), portovenous shunt (from portal vein to hepatic vein) and arterioportal shunt (from hepatic artery to portal vein) [25]. Neurological involvement studies were carried out when neurological symptoms or family history, by a cerebral CT and/or MRI. An endoscopic digestive study was performed when there was disproportionate anaemia to the degree of epistaxis or objectively confirmed overt gastrointestinal (GI) bleeding [7].

### Molecular analysis

All included patients underwent genetic testing. The sequencing technique depended on each treating centers’ preference. Because RiHHTa includes patients from all around Spain, different genetic techniques have been used in the included patients. These techniques include Next generation sequencing (NGS) technologies, Sanger sequencing and other techniques such Multiplex ligation-dependent probe amplification (MLPA). In patients in which NGS was used, coding exons and intronic boundaries of 9 genes related to HHT disease or HHT-like phenotypes (*ACVRL1*, *BMP10*, *BMPR1A*, *BMPR2*, *ENG*, *GDF2*, *RASA1*, *SMAD1*, *SMAD4*) were captured using a custom probe library (SureSelect Target Enrichment Kit for Illumina paired-end multiplexed sequencing method; Agilent Technologies, Santa Clara, California, USA), and sequenced on the HiSeq 1500 platform following Illumina protocols. To determine the pathogenicity of the included variants in the RiHHTa registry, all mutations were checked again by Health in Code SL (A Coruña, Spain) and labeled according to the American College of Medical Genetics and Genomics (ACMG) guidelines [26]. Whenever a genetic variant was identified through NGS, Sanger sequencing was the preferred used method for familial evaluation, unless technically not feasible.

### Statistical analysis

A descriptive statistical analysis was performed for all categorical and continuous variables and expressed as proportions or means with standard deviations (SD), respectively. The descriptive analysis was performed for the entire sample. We used chi-square or Fisher’s exact test to compare categorical data between groups; Fisher’s exact test has been used when expected frequencies were less than 5. Continuous variables were compared using Student t-test. We used two-tailed unpaired t-tests to compare normally distributed continuous data between two groups, and we used the Mann-Whitney U test for non-normally distributed continuous data comparisons. Normality was assessed using Shapiro-Wilk test. *P* values of < 0.05 were considered statistically significant. Analyses were performed using SPSS, version 18 for the PC (SPSS, Inc. Chicago, IL, USA).

## Results

### Baseline characteristics

During the study period, 215 patients from 29 Spanish hospitals were included in the RiHHTa registry, 136 (63.3%) were women. Mean age at time of inclusion of 52.5 ± 16.5 years old. Most (88.4%) patients were Caucasian. The mean age at diagnosis of HHT was 42.2 ± 17.5 years old. Epistaxis was the most frequent symptom at disease onset which allowed the diagnosis of suspicion in 197 (91.6%) patients, while in another 7 (3.2%) patients the diagnosis arose after a central nervous system event (including brain abscess in 4 (1.9%) patients, and stroke, in 3 (1.4%). Family history were present in 178 (82.8%) patients and in 167 (77.7%), muco-cutaneous telangiectasia were observed at diagnosis or during the follow-up. Definite HHT diagnosis defined by the Curaçao criteria were met by 172 (80%) patients, while the remaining were diagnosed through a positive genetic test, after a thorough familial evaluation. .

Epistaxis was also the most frequent symptom at diagnosis of HHT with a mean ESS of 3.65 ± 2.5. Anemia was present in 92 (42.8%) patients, 12 (5%) patients had a history of previous venous thromboembolic disease and 8 (3.7%) patients had atrial fibrillation. Medical attention because of epistaxis at Emergency Department during follow-up was required by 69 (32.1%) patients and red blood cell transfusion by 38 (17.7%) patients.

Pulmonary AVMs were detected in 48 (22.3%) patients and liver VMs identified by imaging test (CT angiography, MRI or doppler ultrasound) were observed in 58 (27%) patients. Pancreatic involvement was detected in seven (3.3%) subjects and other abdominal VMs in 16 (7.4%) patients, including splenic, renal and gastro-omental arterial aneurysms. Brain malformations were reported in five patients (2.3%) and GI telangiectasia were observed in 26 (12.1%) patients (Table 1).

**Table 1:** Baseline characteristics

	n (%) or mean (SD)
Patients, n	215
Sex, n (%)	
Male	79 (36.7)
Female	136 (63.3)
Age at HHT diagnosis (years), mean ± SD	42.2 ± 17.5
Ethnicity	
Caucasian	190 (88.4)
Hispanic	2 (0.9)
Others	9 (4.2)
No clinical data	14 (6.5)
Underlying conditions, n (%)	
Current smoker	26 (12.1)
Hypertension	43 (20)
Diabetes Mellitus	11 (5.1)
Dyslipidemia	33 (15.3)
Venous Thromboembolism	12 (5.5)
Atrial fibrillation	8 (3.7)
HHT criteria, n (%)	
4 HHT criteria	99 (46)
3 HHT criteria	73 (34)
2 or less HHT criteria	32 (14.9)
No clinical data	11 (5.1)
Family history	
Positive	178 (82.8)
Negative	11 (5.1)
No clinical data	26 (12.1)
Symptoms at onset, n (%)	
Nosebleeds	197 (91.6)
Central Nervous system event	7 (3.2)
Cerebral abscess	4 (1.9)
Stroke	3 (1.4)
Cutaneous telangiectases	1 (0.5)
Dyspnea	1 (0.5)
Anemia	1 (0.5)
No clinical data	8 (3.7)
Muco-cutaneous telangiectasia, n (%)	167 (77.7)
Baseline ESS	3.65 (2.5)
Gene mutation, n (%)	
*ENG*	36 (16.7)
*ACVRL1*	77 (35.8)
No clinical data	102 (47.2)
Visceral involvement, n (%)	
Pulmonary AVM	48 (22.3)
Cerebral AVM	5 (2.3)
Hepatic involvement	58 (27)
Gastrointestinal involvement^a^	26 (12.1)

*HHT* Hereditary hemorrhagic telangiectasia, *SD* Standard deviation, *ESS* Epistaxis Severity Score, *AVM* Arteriovenous malformation

^a^Gastrointestinal involvement detected by fibrogastroscopy and / or colonoscopy

### Types of pathogenic variants

**Fig. 1:**
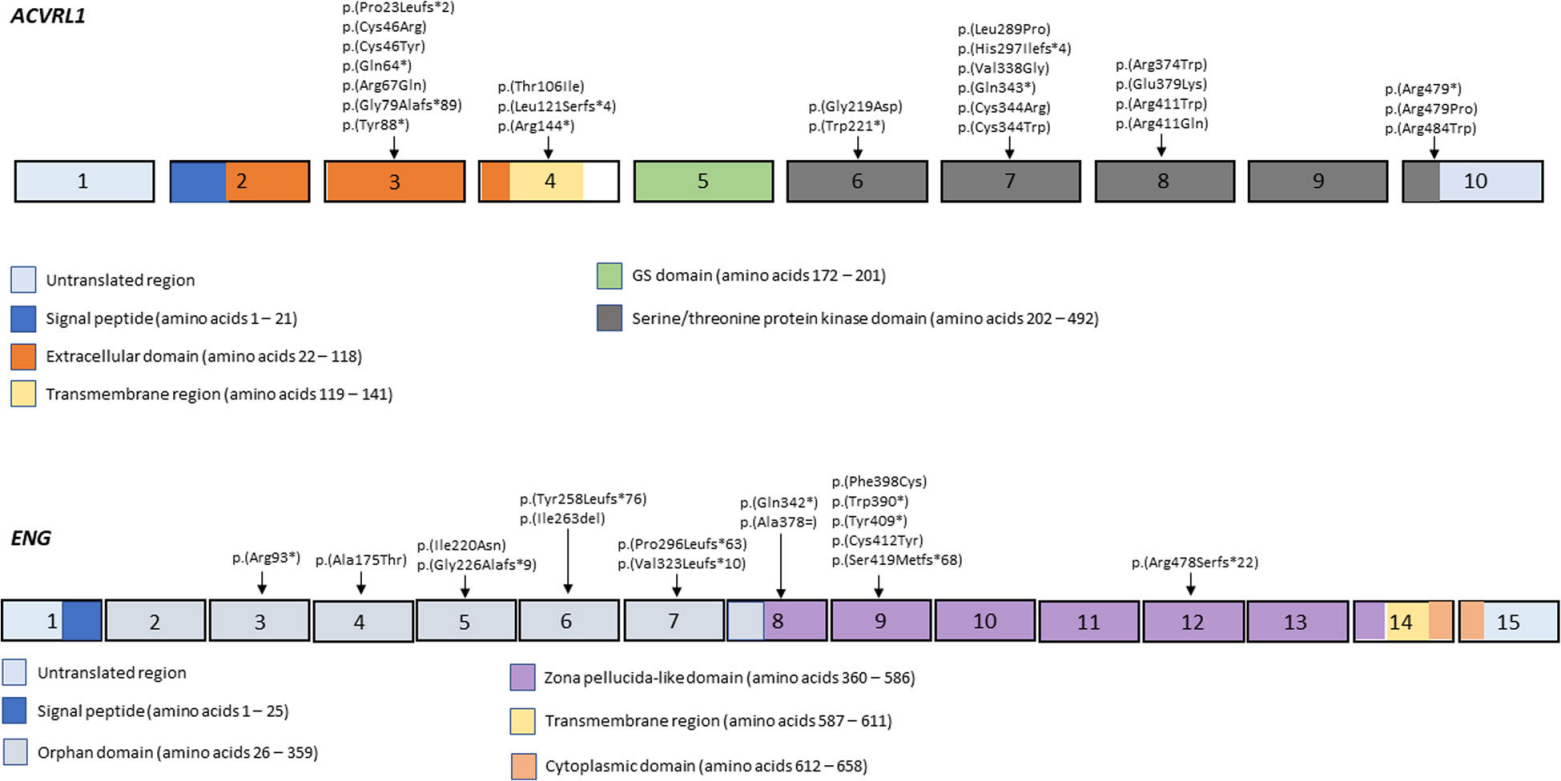
Schematic representation of the exons (boxes) of ACVRL1 and ENG genes. The identified exonic variants in our cohort are presented according to their location (colored boxes represent specific functional domains, each one of them is explained in the legends)

A genetic test was performed in 113 HHT patients from the RiHHTa registry (Table 2). For the mutation analysis, Sanger sequencing was performed in 65 (57.5%) cases, NGS in 38 (33.6%) and other techniques, such as MLPA, in 10 (8.8%) cases. A genetic variant was identified in *ACVRL1* gene in 77 (68.1%) patients, whereas 36 (31.8%) patients harboured a genetic variant in *ENG* gene. According to the type of mutation, *ACVRL1* patients presented more frequently nonsense mutations (50.6%), while in *ENG* patients the most frequent type was frameshift (36.1%). Supplementary Table 1 shows those 52 variants we have complete genetic data, as well as their frequencies in gnomAD and the bioinformatic predictor’s evaluation. Supplementary Table 2 shows the ACMG classification of these variants. Among these variants, we identified 17 missense variants in *ACVRL1*, 41% of them were localized in the kinase domains of exons 7 and 8, and 29% in exon 3. However, missense variants in *ENG* gene were dispersed among the extracellular domain of the protein. In both genes, the predominant variants were the ones that caused loss-of-function mechanism (nonsense, frameshift and splice-site), which would almost certainly be pathogenic in these genes. Figure 1 shows a schematic representation of ACVRL1 and ENG, as well as the exonic variants identified.

**Table 2:** Summary of the variants analysis

GENE mutation	ENG, n (%)	ACVRL1, n (%)
Patients, n	36	77
Sequencing method, n (%)		
Sanger	20 (55.5)	45 (58.4)
NGS	12 (33.3)	26 (33.8)
Others	4 (11.1)	6 (7.8)
Variant type, n (%)		
Nonsense	10 (27.7)	39 (50.6)
Frameshift	13 (36.1)	20 (25.9)
Splice-site	4 (11.1)	3 (3.9)
Missense	9 (25)	15 (19.4)

*NGS* Next-generation sequencing

### Genotype-phenotype correlation

There were no significant differences between *ENG* and *ACVRL1* patients in the age of inclusion, gender, number of Curaçao criteria, recurrent epistaxis, ESS, mucocutaneous telangiectasia, family history, hepatic VMs or GI involvement.

Compared to patients with *ACVRL1* mutations, *ENG* patients were significantly younger at diagnosis (36.9 vs 45.7 years) and usually had more pulmonary AVMs (71.4 vs 24.4%), as well as cerebral AVMs (17.6% vs 2%). Those with *ACVRL1* mutation had a higher cardiac index (3.46 L/min/m^2^ vs 2.62 L/min/m^2^), higher levels of hepatic functional blood tests and more frequently anemia (56.7% vs 28.5%) than *ENG* patients (Table 3).

**Table 3:** Genotype-phenotype correlation in *ENG* and *ACVRL1* patients

GENE Mutation	*ENG*	*ACVRL1*	*p*-value
Patients, n	36	77	
Sex, n (%)			
Male	13 (36)	23 (29.9)	0.507
Female	23 (64)	54 (70.1)	
Age at diagnosis (years), mean ± SD	36.9 ± 17.9	45.7 ± 16.8	0.036
Ethnicity, n (%)			
Caucasian	36 (100)	67 (87)	0.218
Hispanic	0 (0)	5 (6.5)	
Others/unknown	0 (0)	5 (6.5)	
Hypertension, n (%)	8 (22.2)	23 (29.9)	0.133
Diabetes Mellitus, n (%)	1 (2.7)	9 (11.7)	0.146
Dyslipidaemia, n (%)	7 (19.4)	22 (28.5)	0.561
Venous Thromboembolism, n(%)	4 (11.1)	4 (5.2)	0.269
Atrial fibrillation, n (%)	2 (5.5)	6 (7.8)	0.588
HHT criteria, n (%)			0.130
4 HHT criteria	23 (63.8)	38 (49.3)	
3 HHT criteria	9 (25)	27 (35.1)	
2 or less HHT criteria	4 (11.1)	12 (15.6)	
Family history, n (%)			
Positive	34 (94.4)	73 (94.8)	0.626
Negative	1 (2.7)	2 (2.6)	
Unknown	1 (2.7)	2 (2.6)	
Symptoms at onset, n (%)			0.461
Nosebleeds	32 (88.9)	65 (84.4)	
Cerebral abscess	1(2.7)	0 (0)	
Others	2 (5.5)	5 (6.5)	
No clinical data	1 (2.7)	9 (11.7)	
Muco-cutaneous telangiectasia, n (%)	29 (80.5)	62 (80.5)	0.645
Cerebral abscess, n (%)	2 (5.5)	0 (0)	0.132
ESS basal, mean ± SD	3.45 ± 2.5	3.51 ± 2.3	0.94
Anemia, n (%)	10 (27.7)	38 (49.3)	0.026
Cardiac index (L/min/m^2^), mean ± SD	2.62 ± 0.7	3.46 ± 0.79	0.021
Contrast TTE (R-L shunt grade), n (%)			< 0.005
0	2 (5.5)	28 (36.4)	
1	12 (33.3)	17 (22.1)	
2	10 (27.7)	4 (5.2)	
3	4 (11.1)	3 (3.9)	
4	1 (2.7)	0 (0)	
Unknown	1 (2.7)	1 (1.3)	
Pulmonary AVM, n (%)	20 (55.5)	11 (14.3)	< 0.005
Cerebral AVM, n (%)	3 (8.3)	1 (1.3)	< 0.005
Hepatic involvement, n (%)	9 (25)	33 (42.8)	0.075
AV shunt	2 (5.5)	15 (19.4)	0.128
PV shunt	3 (8.3)	2 (2.6)	0.078
AP shunt	1 (2.7)	9 (11.7)	0.385
FNH	0 (0)	2 (2.6)	1
NRH	0 (0)	1 (1.3)	1
Telangiectasia	4 (11.1)	20 (25.9)	0.413
Liver function test (IU/L), mean ± SD			
Aspartate aminotransferase	11.2 ± 10.4	19.7 ± 11.6	0.004
Alanine transaminase	11.5 ± 11.3	19.4 ± 12.5	0.010
Gamma-glutamyl transferase	11.2 ± 12.8	43.4 ± 71.3	0.004
Alkaline phosphatase	35.4 ± 37.1	68.5 ± 69	0.026
Bilirubin	5.61 ± 8.7	5.19 ± 19.7	0.92
Pancreatic involvement, n (%)	0 (0)	6 (7.8)	0.302
Gastrointestinal involvement, n (%)			
Gastroscopy	8 (22.2)	9 (11.7)	0.603
Colonoscopy	3 (8.3)	3 (3.9)	0.622

*AV* Arteriovenous (from hepatic artery to hepatic vein), *PV* Portovenous shunt (from portal vein to hepatic vein), *AP* Arterioportal (from hepatic artery to portal vein). *NRH* Nodular regenerative hyperplasia, *FNH* Focal nodular hyperplasia, *SD* Standard deviation, *R-L* Right-left shunt

## Discussion

The present study using data from the RiHHTa registry represents the largest series about genetic variants of HHT patients in Spain. A previous study reported 74 clinically diagnosed Spanish patients, most of them with *ACVRL1* variants [27]. In our cohort, 77 out of 113 (68.1%) patients also had variants in the *ACVRL1* gene. This finding is in agreement with previous data from other Mediterranean countries but different to Northern Europe or North America, where pathogenetic variants in *ENG* are more frequent than in *ACVRL1* [28, 29].

We have observed that patients with *ENG* gene variants were diagnosed at a younger age. This finding could be influenced because oral and nasal mucosal telangiectases are present earlier in life in patients with HHT1 than with HHT2, so epistaxis also start earlier in these patients [30]. According to previous studies, we have observed a higher prevalence of pulmonary AVMs and cerebral VMs in patients with *ENG* mutations than in those with *ACVRL1* mutations (5–29%) [20, 23, 27, 31]. Hepatic involvement has been described more commonly among *ACVRL1* patients, with a wide range of prevalence from 8 to 78% [29, 30, 32, 33]. In our series, though vascular hepatic involvement was more frequent in *ACVRL1* patients, no statistically significant differences were detected between *ENG* and *ACVRL1* patients, probably because different screening methods were used by RiHHTa investigators. However, patients with *ACVRL1* mutation had a higher CI and levels of hepatic functional blood tests than *ENG* patients, suggesting a more severe hepatic involvement in patients with *ACVRL1* mutations. In fact, in two studies including patients with genotype data available, those who developed symptomatic liver disease had *ACVRL1* mutation [15, 34].

In our study, there were no statistically significant differences in the presence of GI telangiectasia between *ENG* and *ACVRL1* patients. Canzonieri et al. and van Tuyl et al. systemically studied the extent of GI involvement with gastroscopy, video capsule endoscopy, and colonoscopy in 22 and 35 HHT patients, respectively, and found a higher prevalence of telangiectasia in patients with *ENG* mutation [8, 35, 36]. In a recent study, Mora-Luján et al. reported that age, tobacco use, *ENG* mutation, and hemoglobin levels were associated with GI involvement in HHT comparing 67 patients with GI disease and 28 with negative GI study [37]. However, Berg et al., Sabbà et al. and Letteboer et al. assessing genotype-phenotype relationship in HHT patients, reported a similar incidence of GI telangiectasia in HHT1 and HHT2 patients [38–40].

Similar to previous studies, the most frequent type of pathogenic variants we have identified in both *ENG* and *ACVRL1* genes were those predicted to cause a loss-of-function (nonsense, frameshift and splice-site) [19, 24]. Missense variants in *ACVRL1* gene were predominantly localized in the kinase domains of exons 7 and 8, while missense variants in *ENG* gene were dispersed in the extracellular domain of the protein [27]. In a study conducted by Sabbà et al., the correlation between phenotype and variant type was analysed in a cohort of 77 HHT2 patients. *ACVRL1* patients were divided into three subgroups according to the type mutation: truncating mutations (20 patients), missense mutations (47 patients) and the c.626-3C > G splicing mutation (10 patients). Patients with c.626-3C > G mutation had a higher prevalence of pulmonary AVMs than the other two subgroups [29]. This data could be of interest for the evaluation of variants of uncertain significance and for defining useful prediction criteria based on the type of mutation. Genetic study, including the description of new pathogenic variants, usually avoid unnecessary complementary tests, especially in patients with no family history or those who meet less than 3 Curaçao criteria, as young patients [4, 5, 7, 33]. In fact, genetic study has been found preferable to the clinical Curaçao criteria among patients between 0 and 21 years [41].

Our study has some limitations and strengths that should be mentioned. First, it is an observational study from RiHHTa database. Thus, some non-measured variables could lead to a possible bias. Second, genetic and screening tests varied with local practice. Third, a variety of clinicians entered data into the registry, which may lend itself to potential inaccuracies in the data being reported. However the information obtained through RiHHTa registry is valuable and allows thorough understanding the natural history of HHT. Moreover, patients in the RiHHTa registry were from different centers in Spain, which can better reflect epidemiology data of the disease in this country.

## Conclusions

In conclusion, *ACVRL1* variants are most frequent than *ENG* variants in Spain. A description of these identified genetic variants and their clinical significance is provided. Patients with *ACVRL1* variants developed symptomatic liver disease and anemia more usually than *ENG* patients. Compared to *ACVRL1*, those with *ENG* variants were younger at diagnosis and show pulmonary and cerebral AVMs more frequently. The RiHHTa registry can contribute to improve knowledge and management of HHT patients.

## Supplementary information

**Additional file 1: Table S1.** Identified genetic variants in *ACVRL1* and *ENG* genes. **Table S2.** Clinical significance of the identified variants according to ACMG criteria.

## Data Availability

The datasets used and/or analysed during the current study are available from the corresponding author on reasonable request.

## Abbreviations

ACVRL1: Activin A receptor type II-like 1
AVMs: Arteriovenous malformations
CNS: Central nervous system
CT: Computed tomography
ENG: Endoglin
ESS: Epistaxis severity score
GI: Gastrointestinal
HHT: Hereditary hemorrhagic telangiectasia
MLPA: Multiplex ligation-dependent probe amplification
MRI: Magnetic resonance imaging
R-L: Right-left
SD: Standard deviations
TTE: Transthoracic echocardiography
VMs: Vascular malformations
