# Supplementary material for: Current HHT genetic overview in Spain and its phenotypic correlation: data from RiHHTa registry
Source: Orphanet J Rare Dis. 2020 Jun 5;15:138. doi: 10.1186/s13023-020-01422-8 (PMC7275435; doi:10.1186/s13023-020-01422-8)
Supplement: Supplementary file 1 — Additional file 1: Table S1. Identified genetic variants in ACVRL1 and ENG genes. Table S2. Clinical significance of the identified variants according to ACMG criteria. [file 13023_2020_1422_MOESM1_ESM.doc]

**Supplementary Table 1**. **Identified genetic variants in *ACVRL1* and *ENG* genes.**

**ACVRL1 (NM_000020.2)**

| **cDNA** | **Chromosomal**  **location** | | | **Protein** | | **Exon/Intron** | | **gnomAD count** | | **Polyphen2** | | **MutationTaster** | | **CADD** | | **DANN** | | **FATHMM** |
| --- | --- | --- | --- | --- | --- | --- | --- | --- | --- | --- | --- | --- | --- | --- | --- | --- | --- | --- |
| **c.-52G>A** | g.52301433G>A | | |  | |  | | 119/31186 | | - | | - | | 1.158709 | | 0.945977 | | 0.1753 |
| **c.61+1G>T** | g.52306320G>T | | |  | | 2 | | - | | - | | - | | 4.985688 | | 0.995043 | | 0.96194 |
| **c.68delC** | g.52306889delC | | | p.(Pro23Leufs*2) | | 3 | | - | | - | | 1 | | - | | - | | - |
| **c.136T>C** | g.52306957T>C | | | p.(Cys46Arg) | | 3 | | - | | 1 | | 1 | | 5.861518 | | 0.988981 | | 0.93836 |
| **c.137G>A** | g.52306958G>A | | | p.(Cys46Tyr) | | 3 | | - | | 1 | | 1 | | 5.967582 | | 5.967582 | | 0.91811 |
| **c.190C>T** | g.52307011C>T | | | p.(Gln64*) | | 3 | | - | | - | | 1 | | 9.877967 | | 0.997597 | | 0.92124 |
| **c.200G>A** | g.52307021G>A | | | p.(Arg67Gln) | | 3 | | - | | 0.15 | | 0.9999 | | 2.885913 | | 0.997958 | | 0.90047 |
| **c.229T>C** | g.52307050T>C | | | p.(Cys77Arg) | | 3 | | - | | 1 | | 1 | | 3.222914 | | 0.986511 | | 0.8922 |
| **c.236_237delGG** | g.52307057_52307058 delGG | | | p.(Gly79Alafs*89) | | 3 | | - | | - | | 1 | | - | | - | | - |
| **c.264C>G** | g.52307085C>G | | | p.(Tyr88*) | | 3 | | - | | - | | 1 | | 8.616389 | | 0.990752 | | 0.95648 |
| **c.293A>G** | g.52307114A>G | | | p.(Asn98Ser) | | 3 | | - | | 0.91 | | 0.7643 | | 2.995645 | | 0.988443 | | 0.88142 |
| **c.317C>T** | g.52307346C>T | | | p.(Thr106Ile) | | 4 | | 2/233120 | | 0.04 | | 0.9986 | | 2.638408 | | 0.994099 | | 0.58234 |
| **c.360_361insAGCTGGCC** | g.52307389_52307390  insAGCTGGCC | | | p.(Leu121Serfs*4) | | 4 | | - | | - | | 1 | | 2.398303 | | - | | - |
| **c.430C>T** | g.52307459C>T | | | p.(Arg144*) | | 4 | | 1/246414 | | - | | 1 | | 12.310111 | | 0.998291 | | 0.95993 |
| **c.525+2T>C** | g.52307556T>C | | |  | | 4 | | - | | - | | - | | 4.018769 | | 0.986519 | | 0.97156 |
| **c.656G>A** | g.52308253G>A | | | p.(Gly219Asp) | | 6 | | - | | 1 | | 1 | | 6.560594 | | 0.998655 | | 0.99271 |
| **c.663_664delGC** | g.52308260_52308261  delGC | | | p.(Trp221*) | | 6 | | - | | - | | 1 | | - | | - | | - |
| **c.866T>C** | g.52309102T>C | | | p. (Leu289Pro) | | 7 | | - | | 1 | | 1 | | 6.443848 | | 0.999011 | | 0.96983 |
| cDNA | | Chromosomal  location | Protein | | Exon/Intron | | gnomAD count | | Polyphen2 | | MutationTaster | | CADD | | DANN | | FATHMM | |
| c.889delC | | g.52309125delC | p.(His297Ilefs*4) | | 7 | | - | | - | | 1 | | - | | - | | - | |
| c.1013T>G | | g.52309249T>G | p.(Val338Gly) | | 7 | | - | | 1 | | 1 | | 5.887731 | | 0.997058 | | 0.997058 | |
| c.1027C>T | | g.52309263C>T | p.(Gln343*) | | 7 | | - | | - | | 1 | | 13.850123 | | 0.997561 | | 0.97293 | |
| c.1030T>C | | g.52309266T>C | p.(Cys344Arg) | | 7 | | - | | 1 | | 1 | | 5.761432 | | 0.996649 | | 0.95212 | |
| c.1031G>A | | g.52309267G>A | p.(Cys344Tyr) | | 7 | | - | | 1 | | 1 | | 6.592295 | | 0.997329 | | 0.97345 | |
| c.1048+5G>A | | g.52309289G>A |  | | 7 | | - | | - | | - | | 2.141498 | | 0.982146 | | 0.9999 | |
| c.1120C>T | | g.52309891C>T | p.(Arg374Trp) | | 8 | | - | | 1 | | 1 | | 6.358626 | | 0.996962 | | 0.97374 | |
| c.1135G>A | | g.52309906G>A | p.(Glu379Lys) | | 8 | | 2/251236 | | 1 | | 1 | | 6.695163 | | 0.999286 | | 0.99437 | |
| c.1231C>T | | g.52310002C>T | p.(Arg411Trp) | | 8 | | - | | 1 | | 1 | | 8.107635 | | 0.999142 | | 0.98013 | |
| c.1232G>A | | g.52310003G>A | p.(Arg411Gln) | | 8 | | 2/250642 | | 1 | | 1 | | 7.792707 | | 0.999501 | | 0.99439 | |
| c.1378-1G>T | | g.52314542G>T |  | | 9 | | - | | - | | - | | 4.887107 | | 0.993786 | | 0.96675 | |
| c.1435C>T | | g.52314600C>T | p.(Arg479*) | | 10 | | 1/251420 | | - | | 1 | | 13.694196 | | 0.997362 | | 0.92336 | |
| c.1436G>C | | g.52314601G>C | p.(Arg479Pro) | | 10 | | - | | 1 | | 1 | | 7.039674 | | 0.996353 | | 0.96719 | |
| c.1450C>T | | g.52314615C>T | p.(Arg484Trp) | | 10 | | 1/31348 | | 1 | | 1 | | 6.865155 | | 0.999079 | | 0.92497 | |

CADD: Combined Annotation Dependent Depletion; DANN: Deleterious Annotation of genetic variants using Neural Networks; FATHMM: Functional Analysis through Hidden Markov Models; gnomAD: genome aggregation database; Polyphen: Polymorphism Phenotyping

Cut-off values for probable pathogenicity: PolyPhen2: Probably damaging (>=0.909); possibly damaging (>=0.447 - <=0.956); Mutation Taster, DANN and FATHMM: values closer to 1 indicate a high 'security' of the prediction (pathogenicity).

**ENG (NM_001114753.2)**

| cDNA | Chromosomal  location | Protein | Exon/  Intron | gnomAD count | Polyphen2 | MutationTaster | CADD | DANN | FATHMM |
| --- | --- | --- | --- | --- | --- | --- | --- | --- | --- |
| c.361-1G>A | g.130588952C>T |  | 3 | - | - | - | 4.380103 | 0.993834 | 0.94371 |
| c.277C>T | g.130592049G>A | p.(Arg93*) | 3 | - | - | 1 | 11.384597 | 0.997851 | 0.997851 |
| c.523G>A | g.130588789C>T | p.(Ala175Thr) | 4 | - | 0.4 | 0.8 | 4.493035 | 0.997347 | 0.92084 |
| c.659T>A | g.130588004A>T | p.(Ile220Asn) | 5 | - | 0.99 | 0.8321 | 5.995048 | 0.992193 | 0.97983 |
| c.677delG | g.130587988delC | p.(Gly226Alafs*9) | 5 | - | - | 1 | - | - | - |
| c.758T>C | g.130587568A>G | p.(Leu253Pro) | 6 | - | 1 | 1 | 5.051552 | 0.997873 | 0.92533 |
| c.771_772insC | g.130587560_130587  561insG | p.(Tyr258Leufs*76) | 6 | - | - | 1 | 2.437273 | - | - |
| c.787_789delATC | g.130587539_130587  541delTGA | p.(Ile263del) | 6 | - | - | 1 | 1.17072 | - | - |
| c.887delC | g.130587184delG | p.(Pro296Leufs*63) | 7 | - | - | 1 | - | - | - |
| c.967_968delGT | g.130587103_1305871  04delCA | p.(Val323Leufs*10) | 7 | - | - | 1 | 4.543859 | - | - |
| c.1024C>T | g.130586693G>A | p.(Gln342*) | 8 | - | - | 1 | 7.981874 | 0.993636 | 0.21328 |
| c.1134G>A | g.130586583C>T | p.(Ala378=) | 8 | 1/31364 | - | 1 | 1.649539 | 0.90544 | 0.9997 |
| c.1166T>G | g.130582285A>C | p.(Phe389Cys) | 9 | - | 1 | 0.9978 | 4.333362 | 0.989171 | 0.83002 |
| c.1170G>A | g.130582281C>T | p.(Trp390*) | 9 | - | - | 1 | 8.502555 | 0.978717 | 0.29986 |
| c.1227C>G | g.130582224G>C | p.(Tyr409*) | 9 | - | - | 1 | 11.123877 | 0.996971 | 0.39062 |
| c.1235G>A | g.130582216C>T | p.Cys412Tyr | 9 | - | 1 | 0.6976 | 5.878425 | 0.996585 | 0.88865 |
| c.1248_1260delGTCAGCAAGTATG | g.130582191_1305822  03delCATACTTGCTGAC | p.(Ser419Metfs*68) | 9 | - | - | 1 | 7.350449 | - | - |
| c.1434_1435delAG | g.130580652_130580  653delCT | p.(Arg478Serfs*22) | 12 | - | - | 1 | 4.077621 | - | - |
| c.1686+5G>C | g.130580394C>G |  | 12 | - | - | - | 1.553491 | 0.894034 | 0.92863 |

CADD: Combined Annotation Dependent Depletion; DANN: Deleterious Annotation of genetic variants using Neural Networks; FATHMM: Functional Analysis through Hidden Markov Models; gnomAD: genome aggregation database; Polyphen: Polymorphism Phenotyping
Cut-off values for probable pathogenicity: PolyPhen2: Probably damaging (>=0.909); possibly damaging (>=0.447 - <=0.956); Mutation Taster, DANN and FATHMM: values closer to 1 indicate a high 'security' of the prediction (pathogenicity).

gnomAD v2.1.1 (<https://gnomad.broadinstitute.org/>): Accessed on December 15th, 2019

**Supplementary Table 2**. **Clinical significance of the identified variants according to ACMG criteria**

ACVRL1

| **cDNA** | **ACMG** |
| --- | --- |
| c.-52G>A | VUS (PM2, BP4) |
| c.61+1G>T | Pathogenic (PVS1, PM2, PP3) |
| c.68delC | Pathogenic (PVS1, PM2, PP3) |
| c.136T>C | Likely pathogenic (PM1, PM2, PP2, PP3) |
| c.137G>A | Likely pathogenic (PM1, PM2, PP2, PP3) |
| c.190C>T | Pathogenic (PVS1, PM1, PM2, PP3, PP5) |
| c.200G>A | Pathogenic (PM1, PM2, PM5, PP2, PP5, BP4) |
| c.229T>C | Likely pathogenic (PM1, PM2, PM5, PM2, PP3) |
| c.236_237delGG | Pathogenic (PVS1, PM1, PM2, PP3) |
| c.264C>G | Pathogenic (PVS1, PM1, PM2, PP3, PP5) |
| c.293A>G | Likely pathogenic (PM1, PM2, PP2, PP3) |
| c.317C>T | VUS (PM2, PP2, BP4) |
| c.360_361insAGCTGGCC | Pathogenic (PVS1, PM2, PP3) |
| c.430C>T | Pathogenic (PVS1, PM2, PP3, PP5) |
| c.525+2T>C | Pathogenic (PVS1, PM2, PP3) |
| c.656G>A | Likely pathogenic (PM1, PM2, PP2, PP3, PP5) |
| c.663_664delGC | Pathogenic (PVS1, PM1, PM2, PP3) |
| c.866T>C | Likely pathogenic (PM1, PM2, PP2, PP3, PP5) |
| **c.889delC** | Pathogenic (PVS1, PM1, PM2, PP3, PP5) |
| **c.1013T>G** | Likely pathogenic (PM1, PM2, PP2, PP3) |
| **c.1027C>T** | Pathogenic (PVS1, PM1, PM2, PP3, PP5) |
| **c.1030T>C** | Pathogenic (PS1, PM1, PM2, PM5, PP2, PP3, PP5) |
| **c.1031G>A** | Pathogenic (PM1, PM2, PM5, PP2, PP3, PP5) |
| **c.1048+5G>A** | VUS (PM2, BP4) |
| **c.1120C>T** | Pathogenic (PM1, PM2, PM5, PP2, PP3, PP5) |
| **c.1135G>A** | Pathogenic (PM1, PM2, PP2, PP3, PP5) |
| **c.1231C>T** | Pathogenic (PM1, PM2, PP2, PP3, PP5) |
| **c.1232G>A** | Pathogenic (PM1, PM2, PM5, PP2, PP3, PP5) |
| **c.1378-1G>T** | Pathogenic (PVS1, PM2, PP3) |
| **c.1435C>T** | Pathogenic (PVS1, PM1, PM2, PP3, PP5) |
| **c.1436G>C** | Likely pathogenic (PM1, PM2, PM5, PP2, PP3) |
| **c.1450C>T** | Pathogenic (PM1, PM2, PM5, PP2, PP3, PP5) |

ENG

| **cDNA** | **ACMG** |
| --- | --- |
| **c.361-1G>A** | Pathogenic (PVS1, PM2, PP3) |
| **c.277C>T** | Pathogenic (PVS1, PM1, PM2, PP3, PP5) |
| **c.523G>A** | Likely pathogenic (PM1, PM2, PM5, PP2, BP4) |
| **c.659T>A** | Likely pathogenic (PM1, PM2, PM5, PP2, PP3) |
| **c.677delG** | Pathogenic (PVS1, PM1, PM2) |
| **c.758T>C** | Likely pathogenic (PM1, PM2, PP2, PP3) |
| **c.771_772insC** | Pathogenic (PVS1, PM1, PM2, PP3, PP5) |
| **c.787_789delATC** | Likely pathogenic (PM1, PM2, PM4, PP3) |
| **c.887delC** | Pathogenic (PVS1, PM1, PM2) |
| **c.967_968delGT** | Pathogenic (PVS1, PM1, PM2, PP3) |
| **c.1024C>T** | Pathogenic (PVS1, PM2, PP5) |
| **c.1134G>A** | Likely pathogenic (PM1, PM2, PP5, BP4) |
| **c.1166T>G** | VUS (PM1, PM2, PP2, BP4) |
| **c.1170G>A** | Pathogenic (PVS1, PM1, PM2) |
| **c.1227C>G** | Pathogenic (PVS1, PM1, PM2) |
| **c.1235G>A** | Likely pathogenic (PM1, PM2, PM5, PP2, PP3) |
| **c.1248_1260delGTCAGCAAGTATG** | Pathogenic (PVS1, PM1, PM2, PP3) |
| **c.1434_1435delAG** | Pathogenic (PVS1, PM1, PM2, PP3) |
| **c.1686+5G>C** | VUS (PM2, BP4) |

American College of Medical Genetics and Genomics (ACMG); BP: bening supporting; PM: pathogenic moderate; PP: pathogenic supporting; PS: pathogenic strong; PVS: pathogenic very strong; VUS: variant of unknown significance.
